# The World Hypertension League Science of Salt: a regularly updated systematic review of salt and health outcomes studies (Sept 2019 to Dec 2020)

**DOI:** 10.1038/s41371-022-00710-z

**Published:** 2022-06-10

**Authors:** Nan Xin Wang, JoAnne Arcand, Norm R. C. Campbell, Claire Johnson, Daniela Malta, Kristina Petersen, Sarah Rae, Joseph Alvin Santos, Bridve Sivakumar, Sudhir Raj Thout, Rachael McLean

**Affiliations:** 1 Department of Preventive and Social Medicine, University of Otago, 18 Frederick St, Dunedin, 9016 New Zealand; 2 Faculty of Health Science, Ontario Tech University, Oshawa, ON Canada; 3 Department of Medicine, Physiology and Pharmacology and Community Health Sciences, Libin Cardiovascular Institute of Alberta, University of Calgary, Calgary, AB Canada; 4 The George Institute for Global Health, University of New South Wales, Sydney, NSW Australia; 5 School of Nutrition, Ryerson University, Toronto, ON Canada; 6 Department of Nutritional Sciences, Texas Tech University, Lubbock, TX USA; 7 Department of Nutritional Sceinces, Faculty of Medicine, University of Toronto, Toronto, ON Canada; 8 The George Institute for Global Health, Hyderabad, India

**Keywords:** Risk factors, Hypertension

## Abstract

The World Hypertension League Science of Salt health outcomes review series highlights high-quality publications relating to salt intake and health outcomes. This review uses a standardised method, outlined in previous reviews and based on methods developed by WHO, to identify and critically appraise published articles on dietary salt intake and health outcomes. We identified 41 articles published between September 2019 to December 2020. Amongst these, two studies met the pre-specified methodological quality criteria for critical appraisal. They were prospective cohort studies and examined physical performance and composite renal outcomes as health outcomes. Both found an association between increased/higher sodium intake and poorer health outcomes. Few studies meet criteria for high-quality methods. This review adds further evidence that dietary salt reduction has health benefits and strengthens evidence relating to health outcomes other than blood pressure and cardiovascular disease. We observe that most studies on dietary sodium do not have adequate methodology to reliably assess sodium intake and its association with health outcomes.

## Introduction

Detrimental effects of high salt intake on blood pressure (BP), especially systolic BP, are well documented in the literature [1–3]. High BP, a primary modifiable risk factor for cardiovascular diseases (CVD) [4], can be prevented and managed with reduced salt intake [5]. For this reason, the World Health Organization (WHO) recommends intakes of less than 5 g/day of salt, or 2 g/day of sodium, in adults for chronic disease prevention [3].

Recently, the United States of America (USA) and Canada’s Dietary Reference Intakes for sodium were updated based on an extensive review of the evidence on sodium intake and chronic disease risk [6]. It recommended chronic disease risk reduction intakes to be below 2300 mg sodium /day based on clear evidence relating to BP and CVD. Associations with other health effects, such as incident chronic kidney diseases (CKD), osteoporosis and all-cause mortality were also examined; however, there were few studies of adequate quality that provided strong evidence to suggest risk reduction when sodium intake is lowered. The Committee concluded that more studies of high methodological quality is required to study the effects of salt intake on health outcomes.

Recent controversy surrounds claims by some authors that salt intakes below 7.5 g/day (3000 mg sodium) may be associated with increased risk of adverse health outcomes [7–9]. This is in contrast to recommendations from WHO [10] and other organisations [11, 12] to reduce dietary salt intake to below 5 g/day (2000 mg sodium) for individuals and populations. Some recent observational research has shown a J shaped curve when dietary salt intake is associated with cardiovascular disease outcomes, suggesting that lower salt intake is associated with higher risk [13, 14]. However inaccurate measurement of salt intake at baseline (using spot urine) has been shown to be a feature of these studies [15, 16]. Other methodological issues contribute to controversies in interpreting evidence relating to salt intake. For example, due to the ubiquity of salt in the food supply high-quality randomised controlled trials often fail to achieve long-term meaningful differences in salt intake between groups. Observational studies may be subject to reverse causality and residual confounding.

For these reasons, we have adapted criteria developed by WHO [10] and the Agency for Healthcare Research and Quality U.S. Department of Health and Human Services evidence review [11, 17] to assess study quality in these Science of Salt health oucomes systematic reviews. This article aims to identify and critically appraise studies that are of high methodological quality published between September 2019 to December 2020 that relate to salt intake and health outcomes.

## Methodology

A standard methodological approach has been taken with all Science of Salt systematic reviews. A detailed description of this standard approach has been previously published [18], and is summarised below. Briefly, a standard search strategy is used to identify articles that meet our inclusion criteria over a set period. We aim to identify articles that examine the effect of dietary salt intake on outcomes meaningful to people and patients. We highlight and critically review studies that meet pre-specified criteria for being of high quality.

A standard MEDLINE search strategy is used to identify articles for all reviews. The search strategy was adapted from a systematic review used by WHO to develop the guideline: Sodium intake for adults and children (2012), and the National Academy of Science Dietary Reference Intakes for Sodium (2019) [3, 11, 19], and has been standardised to be used in the Science of Salt review series [18]. Articles are included if they were, i. human research, ii. original investigations, iii. assessed salt intake, and iv. assessed a health related or relevant surrogate outcome. All study designs were considered. Studies were excluded if they were i. animal or in-vitro studies, ii. narrative reviews, commentaries, protocols, position papers, case reports, letters to the editor, proceedings, or guidelines or iii. studies in which dietary salt is not the only exposure variable. Subsequently, for assessment in the detailed critical appraisals, articles were screened based on pre-specified criteria for health outcomes examined and methodological quality (Tables S1 and S2). This was conducted by two researchers independently. Here we include published articles identified between September 2019 to December 2020.

Health outcomes were ranked based on their relevance to patients’ experiences, with greatest importance given to mortality (Category I) and morbidity (Category II), followed by symptoms/quality of life/functional status (Category III), clinical surrogate outcomes (Category IV) and other clinical surrogate outcomes (Category V). By contrast, physiological/biomarker surrogate outcomes (Category VI) were excluded from the detailed critical appraisal as they were considered as less important to patients. The studies also had to meet methodological quality criteria to be considered for detailed critical appraisal. These studies were randomised controlled trials (RCT) with a control group and an intervention group with altered (high or low) sodium intake, achieved an intake difference of ≥920 mg sodium (2.3 g of salt) between the intervention and control, were ≥4 weeks in duration for studies in Category III and IV outcomes or ≥ 1 year for studies in Category I, II and, V, measured sodium intake with at least one 24-h urine collection, and had no concomitant interventions (i.e., hypertensive drugs, other dietary interventions). Cohort studies were included if they were of ≥1 year in duration for studies in Category I, II and, V, and ≥4 weeks in duration for studies in Category III; had ≥400 participants (continuous outcomes) or events (dichotomous outcomes); and measured sodium intake with at least one 24-h urine collection, food record or 24-h diet recall [20, 21]. Where a food frequency questionnaire (FFQ) was used to assess sodium intake, the study was excluded because FFQ was found to be in poor agreement with 24-h urine collection for estimation of sodium intake by a systematic review [22]. Studies that estimated sodium intake using spot urine (or urine collections for less than a 24 h period) were excluded from detailed critical analysis because spot urine has been shown to be an inaccurate measure of individual sodium intake [15, 23]. For cohort studies, sodium intake or excretion alone had to be the exposure variable. Articles that only included sodium-to-potassium ratio or related variables were not eligible for detailed critical appraisal. Meta-analyses of RCTs or cohort studies had to include studies of the same criteria defined for their respective designs. Cross-sectional studies were excluded from detailed critical appraisals.

Detailed critical appraisals for risk of bias were performed independently by two researchers, and any discrepancies were discussed with the wider research team. A modified risk of bias tool for observational, non-randomised studies was used [24]. Articles not included in the detailed critical analysis are still reviewed and may be discussed, especially if they highlight particular issues of methodological relevance.

This initiative is supported by the George Institute for Global Health, the World Hypertension League, the WHO Collaborating Centre on Population Salt Reduction, the Pan American Health Organization/WHO Technical Advisory Group on Cardiovascular Disease Prevention through Dietary Sodium, and the World Action on Salt & Health.

## Results

**Fig. 1:**
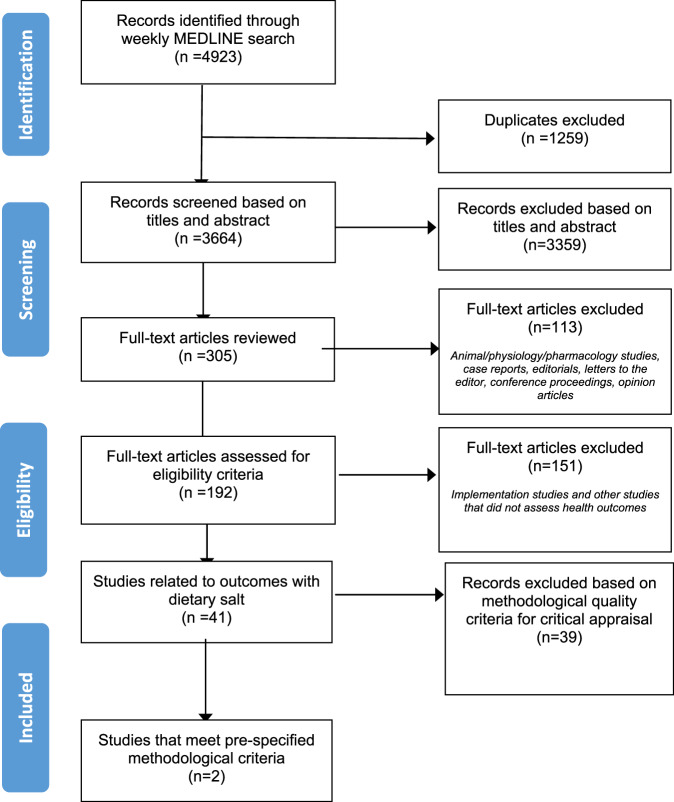
PRISMA study flow diagram for studies identified from September 2018 to December 2020.

The weekly search identified 4923 citations, of which 192 studies were eligible for full-text review (Fig. 1). Table 1 shows the 41 studies that were found to be related to dietary salt and health outcomes. The studies included were meta-analysis (*n* = 1), RCT (*n* = 12), quasi-experimental study (*n* = 1), intervention study (*n* = 3), prospective cohort study (*n* = 7), cross-sectional (*n* = 16), and post-hoc analysis of prospective cohort study (*n* = 1). Amongst the studies, a variety of outcomes were assessed. Category I mortality and incident CVD [25], Category II composite CVD [26] and Category III with the outcome of physical performance [27] had one study each. Category IV included the largest number of studies (*n* = 19), all of which assessed BP outcomes [28–46]. Two studies assessed Category V outcomes (composite renal outcome [47] and fatty liver index [48]). A further eighteen studies assessed Category VI outcomes such as plasma volume, estimated glomerular filtration rate and albuminuria [49–65].

**Table 1:** Summary of 41 studies identified from September 2019 to December 2020 and eligibiltiy for full-text review.

	Study (Country)	Study design	Study duration	Method of sodium intake measurement	Outcomes	Eligible for detailed critical appraisal	Reson for exclusion from detailed critical appraisal
	**Category I—Mortality outcomes**						
1	Elliot et al., United Kingdom [25]	Prospective cohort study	Median follow-up 5 years	Spot urine	Mortality and incident cardiovascular disease	No	Spot urine
	**Category II - Morbidity outcomes**						
2	Sadanaga et al., Japan [26]	Post hoc (sub-group) analysis of a prospective cohort study	5.2 years	Spot urine	Compositie CVD events (Heart failure, hospitalisation, acute coronary syndrome)	No	Spot urine
	**Catorory III - Symptoms/ Quality of life/Functional status outcomes**						
3	Lana et al., Spain [27]	Prospective cohort study	5 years	Diet recall (history)	Physical performance	Yes	
	**Category IV - Clinical surrogate outcomes (Blood pressure)**						
4	Arantes et al., Brazil [28]	Intervention study (no control)	5 months	24 h urine	Blood pressure (central and ambulatory)	No	Intervention study- no control group. No change in sodium intake assessed by 24 h urine excretion
5	Arvizu et al., Denmark [29]	Prospective cohort study	Duration of pregnancy	FFQ	Hypertensive disorders of pregnancy	No	FFQ, short-term follow-up
6	Babcock M, United States [30]	RCT cross-over	10 days	24 h urine	Exercise induced blood pressure	No	RCT less than 4 weeks
7	Bovee et al., Netherlands [31]	RCT cross-over	2 weeks	24 h urine	Blood pressure, kidney function and fluid balance	No	RCT less than 4 weeks
8	Chaudhary et al., United States [32]	RCT cross over (reanalysis of DASH sodium trial data)	4 weeks	24 h urine	Systolic blood pressure	No	Examined sodium and potassium ratio as exposure variable
9	Elfassy et al., United States [33]	Cross sectional	N/A	24 h urine	Blood pressure control among individuals with hypertension	No	Cross sectional
10	Han et al., China [34]	Cross sectional	N/A	24 h urine	Blood pressure	No	Cross sectional
11	Humalda et al., Netherlands [35]	Randomsied controlled trial	3 month intervention plus 6 months maintenance	24 h urine	Blood pressure, proteinuria, costs, quality of life, self management skills	No	Difference in sodium intake too small (28 mmol/day after 3 months and no difference after maintenance phase)
12	Kolahdooz et al., Canada [36]	Cross sectional	N/A	24 h diet recall	Blood pressure	No	Cross-sectional study design
13	Kwon et al., Republic of Korea [37]	Cross sectional	N/A	Semi-quantitative FFQ	Blood pressure	No	Semi-quantitative FFQ for sodium assessment
14	Li et al., United States [38]	Cross sectional	N/A	Two 24 h recalls	Blood pressure	No	Cross-sectional study design
15	Lin et al., China [39]	Cross sectional	N/A	FFQ	Blood pressure	No	Cross-sectional study design, FFQ
16	Migdal, Babcock et al., United States [40]	Crossover RCT (feeding study)	10 days each	24 h urine	Blood pressure variability	No	Study duration <4 weeks
17	Migdal, Robinson et al., United States [41]	Crossover RCT (single meal study)	Two single days, separated by 1 week	Not measured- meal composition was exposure variable	Postprandial, post-exertion blood pressure response	No	Study duration <4 weeks
18	Naser et al., Bangladesh [42]	Validation study		24 h urine and spot urine	Blood pressure	No	Cross-sectional study design
19	Panuccio et al., Italy [43]	Randomised controlled trial	6 months	24 h urine	Blood pressure, 24 h urinary sodium	No	Implementation study: Tested urinary chloride strips to see if they promoted HTN control and Na adherence in a CKD population
20	Reynoso-Marreors et al., Peru [44]	Quasi-experimental study	8 weeks, (4 week intervention)	Not measured- meal composition	Blood pressure	No	No measure of sodium intake, no control group
21	Shenouda et al., United States [45]	Cross over RCT (feeding study)	14 days total (7 days per diet)	24 h urine	Blood pressure and low-flow mediated vascular constriction	No	Duration <4 weeks
22	Sougawa et al., Japan [46]	Cross sectional	N/A	FFQ	Blood pressure	No	Cross sectional
	**Category V - Clinical surrogate outcomes (Other)**						
23	Kang et al., Republic of Korea [47]	Prospective cohort study	Median follow-up 4.3 years	24 h urine	Composite renal outcome, which was defined as either halving the estimated glomerular filtration rate or developing end-stage renal disease.	Yes	
24	Van den Berg et al., Netherlands [48]	Cross sectional analysis of a prospecitve cohort	N/A	Two 24 h urine	Fatty liver index, hepatic steatosis	No	Cross-sectional study design
	**Category VI – Physiologic surrogate outcomes**						
25	Adolf et al., Germany [49]	Clinical cohort study	3 years	24 h urine	Salt intake	No	Oucome salt intake, fewer than 400 patients (n = 323)
26	Babcock M, United States [50]	RCT cross-over	10 days	24 h urine	Plasma volume, post exercise	No	RCT less than 4 weeks
27	Baldo et al., Brazil [51]	Cross sectional	N/A	12 h urine	Arterial stiffness (carotid‐femoral pulse wave velocity)	No	Cross sectional, 12 h urine
28	Bossola et al., Italy [52]	Cross sectional	N/A	3 day diet record	Intake of other nutrients	No	cross sectional, outcome was associated nutrient intake, not health outcome
29	Braconnier et al., Swizerland [53]	Intervention study, randomised, cross over	5 days	24 h urine	Sweat and muscle sodium	No	Intervention study, 5 days intervention period
30	Burgermaster et al., N/A [54]	Systematic review and meta-analysis: RCTs and cohort studies	Varied	24 h urine, self report and 3 day diet record	Various	No	Not health outcomes
31	Chen et al., United States [55]	Cross over study	5–7 days	24 h urine	Mean 24 h urinary free cortisol	No	Short intervention period
32	Chen et al., United States [56]	RCT cross-over	6 weeks	No measure sodium intake	Circulating short-chain fatty acids	No	No measure of sodium intake
33	Choi et al., Republic of Korea [57]	Observational cross sectional	N/A	Spot urine	Triglycerides	No	Cross sectional, spot urine
34	Jardine et al., China [58]	Cluster randomised controlled trial	18 months	24 h urine	Albuminuria	No	Difference between groups only 14 mmol/day
35	Kramers et al., Netherlands [59]	Prospective cohort study	4 years	24 h urine at baseline and weeks 12, 48, 96, 120 and 132	eGFR	No	Category VI outcome (physiologic surrogate outcome)
36	Lobene et al., United States [60]	Validation study	N/A	Spot urine	Urinary sodium	No	Spot urine/ validation study
37	Martinelli et al., Brazil [61]	Cross sectional	N/A	24 h urine and an FFQ	Salt taste sensitivity	No	Cross-sectional study design
38	Petermann-Rocha et al., Chile [62]	Cross sectional	N/A	Spot urine	Sociodemographic variables	No	Cross-sectional study design, spot urine
39	Wright et al., Australia [63]	Cross sectional	N/A	24 h urine	Behavioural factors associated with sodium intake	No	Cross-sectional study design
40	Wenstedt et al., Netherlands [64]	Cross over RCT	16 days (8 days per diet)	Six 24 h urine (three per diet)	Extracellular fluid volume	No	Study period <4 weeks
41	Yoshimura et al., Japan [65]	Prospective cohort study	1 year	Spot urine	Albuminuria	No	Spot urine intake

Eleven RCTs were excluded from detailed critical appraisal because they were less than four weeks for category III and IV outcomes (*n* = 9), or less than 2.3 g/day difference of salt intake (920 mmol/day of sodium) (*n* = 2). Five prospective cohort studies were ineligible because they used low-quality methods to measure dietary sodium (spot urine: *n* = 3; FFQ: *n* = 1) or had fewer than 400 participants in the study (*n* = 1). A main reason for exclusion was weak study design, for example, cross sectional (*n* = 16), quasi-experimental (*n* = 1) and non-controlled intervention study (*n* = 1). Other reasons for exclusion included dietary sodium not an exposure (*n* = 2), dietary sodium not measured (*n* = 1), Category VI physiological surrogate outcome (*n* = 1) and, meta-analysis that did not measure health outcomes (*n* = 1).

Finally, two prospective cohort studies were critically appraised [27, 47]. The Seniors-ENRICA cohort (mean age 70.5 years old) included community-dwelling individuals in Spain. Using face-to-face diet history to assess dietary sodium, they found that increased sodium intake over five years was associated with decreased physical performance. Adjusted models showed similar associations [27]. The second study consisted of participants from the KNOW-CKD cohort in Korea. The study had a median follow-up of 4.3 years. The third and fourth quartiles of measured 24-h urinary sodium excretion were associated with higher risk of developing composite renal outcomes compared to the second quartile (adjusted hazard ratio1.69 (95% CI 1.08–2.65) and 1.80 (95% CI 1.12–2.88) respectively) [47].

A summary of the study characteristics, results and risk of bias assessment are presented in the section below. A complete reasoning for the scores given in the risk of bias assessment for the two studies included for detailed critical appraisal is in Table S3.

## Studies that met the minimum methodological quality criteria

1. What is the effect of salt intake on physical performance in free-living adults?

Lana A, Struijk EA, Ortolá R, Rodríguez-Artalejo F, Lopez-Garcia E. Longitudinal Association Between Sodium and Potassium Intake and Physical Performance in Older Adults. The Journals of Gerontology: Series A. 2020;75(12):2379–86.

*Design:* prospective cohort study

*Setting:* Spain, the Seniors-ENRICA cohort

*Follow-up period:* 5 years, data collected at two time points, 2012 and 2017

*Participants:* stratified cluster sampling of individuals in Spain. Sub-sample of ≥ 60 years old with follow-up rate of 62.1% (*n* = 1130). Final sample, *n* = 868 community-dwelling individuals ≥ 60 years old. Mean age 70.5 years at baseline, 49.1% male.

*Exposure:* changes in sodium intake, estimated by computer-assisted face-to-face diet history of 880 foods and beverages in the previous year.

*Outcomes:* changes in scores of Short Physical Performance Battery (SPPB). SPPB evaluates lower extremity function and was developed by the National Institute on Aging [66]. This is one of the most widely used test for physical function in elderly [67] and has also been proven to be valid and reliable in several populations [68, 69]. Multivariable linear regression to assess the association of changes in sodium intake and overall SPPB scores and its individual components (standing balance, gait speed and chair stand). Each component is awarded a maximum of 4 points, combined score of 12 points indicates best performance while 0 point is the worst performance.

## Risk of bias

- *Sampling:* low
- *Representativeness:* high
- *Reliability/validity of exposure:* high
- *Reliability/validity of outcome:* low
- *Blinding of outcome assessment:* low
- *Risk of selective outcome reporting:* low
- *Confounding:* low

*Source of funding:* Fondo de Investigaciones Sanitarias, Instituto de Salud Carlos III, the ATHLOS project and the SALAMANDER project.

## Summary of results

Overall, sodium intake between the two surveys (2012 and 2017) reduced from a mean of 2.68 g/day (SD 9.56) to 2.53 g/day (SD 8.21). The SPPB score also declined from 8.86 (SD 2.16) to 8.77 (SD 2.65). The authors examined the association between changes in sodium intake and SPPB scores between the two surveys (i.e., positive number means increase in sodium intake or an improvement in physical function). A decrease in sodium intake (tertile 1, mean change −1.06 g/day) showed an increase in SPPB score of 0.06 (95% CI − 0.30; 0.41) when compared to little to no change in sodium intake (tertile 2, mean change −0.15 g/day). An increase in sodium intake (tertile 3, mean change 0.74 g/day), in comparison with tertile 2 was associated with a 0.45 (95% CI −0.81: −0.09) decrease in the SPPB score over the survey period. Every SD (0.73 g/day sodium) increase in sodium intake was associated with a 0.13 (95% CI −0.26; −0.01) reduction in SPPB score. The linear dose-response relationship was similar across the adjusted models. A reduction in the chair stand test score was most associated with an increase in sodium intake.

## Comment

This prospective analysis of the Seniors-ENRICA cohort found that among older adults an increase in dietary sodium intake over a five-year period was associated with lower physical performance. The strengths of this study include its prospective design, sampling strategy used, and measurement of dietary sodium at two different time points using diet history. However, there are also some limitations. The participants included in this study had better health indicators than the original Seniors-ENRICA cohort at baseline, thereby reducing the external validity of current findings. There is also a lack of data on incidence of chronic kidney disease in the sample, which is associated with dietary changes related to sodium. Dietary intake was measured using a face-to-face diet history, which can introduce social desirability and recall bias, leading to misreporting and measurement error when estimating mean sodium intake. In fact, the authors state that the diet history did not include discretionary salt use during cooking and at the table. In a previous validation study, the authors showed that the diet history had a moderate (*r* = 0.56) and weak (*r* = 0.19) correlation strength when compared with sodium intake estimated using 24-h diet recalls and 24-h urinary sodium respectively [70]. However, a previous meta-analysis of studies comparing 24-h diet recall and 24-h urine for assessment of sodium intake in individuals has shown that 24-h diet recall is not accurate, it is recommended that individual sodium intake should not be assessed via 24-h diet recall [20]. We have therefore scored the reliability/validity of exposure as high. Moreover, as discussed by the authors, there is potential for reverse causality as physical impairment may decrease participants’ ability to acquire and to cook healthy diet that is lower in sodium. Survivor bias cannot be excluded with incomplete follow-up.

We have scored confounding as low because the authors has collected data on potential confounders and justified their inclusions in the three different models that were fitted. Nonetheless, we cannot elimitate the possibility of residual confounding because it is an obserational study.

2. Does dietary salt intake affect renal outcomes in chronic kidney disease patients?

Kang M, Kang E, Ryu H, Hong Y, Han SS, Park SK, et al. Measured sodium excretion is associated with CKD progression: results from the KNOW-CKD study. Nephrology Dialysis Transplantation. 2020;36(3):512–9.

*Design:* prospective cohort study

*Setting:* nine tertiary hospitals in Korea, KoreaN cohort study for Outcome in patients With Chronic Kidney Disease (KNOW-CKD) cohort

*Follow-up period:* median follow-up period 4.3 (IQR: 2.8–5.8) years

*Participants:* *n* = 1254, patients with Chronic Kidney Disease stages 1–5 (pre-dialysis) aged between 20 and 75 years old at baseline. Median age 58.0 (IQR: 50.0–66.0) years old, 62% male.

*Exposure:* 24-h urinary sodium excretion at baseline

*Outcomes:* End-Stage Renal Disease (ESRD) or estimated Glomerular Filtration Rate (eGFR) halving. Hazard ratio of the outcome was analysed using Cox proportional hazards models. Association of urinary excretion and CKD progression explored using cubic spline curves.

## Risk of bias

- *Sampling:* high
- *Representativeness:* unclear
- *Reliability/validity of exposure:* low
- *Reliability/validity of outcome:* low
- *Blinding of outcome assessment:* low
- *Risk of selective outcome reporting:* low
- *Confounding:* Low

*Source of funding:* Korea Centers for Disease Control and Prevention

## Summary of results

Higher sodium intake in the third (3.34–4.44 g/day) and fourth quartile (≥4.44 g/day) is associated with higher risk of composite renal outcomes (Hazard Ratio (HR) 1.69, 95% CI 1.08–2.65; HR 1.80, 95% CI 1.12–2.88 respectively) compared with the second quartile (2.40–3.34 g/day). There is no difference in risk of composite renal outcomes in the first quartile of sodium intake (<2.4 g/day) compared with the second quartile of intake (HR 1.28, 95% CI 0.84–1.98). For participants with urinary sodium excretion of ≥2.76 g/day, there was a linear association of 55% increase in risk of composite renal outcomes for every 2.3 g increase in sodium excretion. Sub-group analyses comparing the highest quartile of 24 h sodium urinary excretion to second quartile showed higher risk of CKD in those who were at baseline: under 60 years of age (HR 2.19, 95% CI 1.16–4.16), females (HR 6.41, 95% CI 2.54–16.21), with lower eGFR (<45 mL/min/1.73 m^2^) (HR 2.22, 95% CI 1.32–3.71), with uncontrolled hypertension (SBP  ≥130 mmHg or DBP ≥80 mmHg) (HR 3.49, 95% CI 1.32–9.25), who were overweight/obese (BMI≥25 kg/m^2^) (HR 2.34, 95% CI 1.05–5.19), used RAS blocker (1.75, 95% CI 1.06–2.89) and with 24-h urinary potassium of ≥1.45 g/day (HR 2.29, 95% CI 1.15–4.59).CKsD.

## Comment

In this large, nationwide prospective cohort study of patients with CKD, higher sodium intake was associated with increased risk of composite renal outcomes. The sub-group analyses identified those at higher risk of CKD progression due to high salt intake. The strengths of the study are the prospective design, long follow-up period and the use of 24-h urine to quantify sodium intake at baseline. However, there are also limitations. Only one 24-h urine collection was conducted at baseline over the whole study period. While 24-h urine is a high-quality method for measuring sodium intake, one measurement may not be sufficient to estimate individual intake. Multiple 24-h urine collections are needed to prevent misclassification and establish reliable associations of health risks and sodium intake [71]. It is also unclear if sodium intake has changed over-time because of disease progression or intrinsic motivation. Another limitation is the non-random sample that was recruited in the study. There is no information on the response rate or comparison to the underlying population to assess the representativeness of the study participants. The risk of bias score for confounding was rated as low because study adjusted for known confoundings which were appropriately included in three different models. Despite the adjustments made, the risk of residual confounding remains due to the observational nature of the study design.

## Discussion

As with previous Science of Salt reviews, we found very few high-quality studies examining the relationship between salt intake and important health outcomes. This Science of Salt health outcomes review identified 41 studies published between September 2019 to December 2020. A majority of the studies assessed BP or CVD-related outcomes (22 out of 41), however, only two studies met the minimum methodological quality criteria for detailed critical appraisal. The two studies were prospective cohort in design, and assessed physical performance [27] and composite renal outcomes [47]. Overall, these studies showed that increased or higher sodium intake is associated with poorer health outcomes.

In the series of Science of Salt health outcomes reviews [72–80], this is the first to have identified physical performance as a health outcome. This is a valuable addition to the scope of adverse health outcomes relating to high sodium intake as it addresses an important quality of life indicator. High sodium intake has been associated with increased urinary excretion of calcium, lower bone mineral density, reduced muscle strength and sarcopenia [81, 82]. These factors contribute to physical performance. Lana and colleagues used the Short Physical Performance Battery (SPPB) test to measure physical performance. This method is rated highly for validity, reliability and responsiveness [67]. Studies have found that lower scores on the SPPB are associated with an increased risk of falling, loss of independence to perform daily activities, decreased mobility and death [83]. Although we agree that physical performance is an important aspect of health to investigate, the method of sodium intake assessment, diet history, has high risk of bias. The use of multiple 24-h urine samples to estimate sodium intake would have strengthened this field of study.

The second study that was included in our detailed critical analysis adds further high-quality evidence, demonstrating the association between chronic kidney diseases and sodium intake. The 2019 National Academies of Sciences, Engineering, and Medicine committee reviewing the dietary reference intakes for sodium and potassium for the USA and Canada, stated in their report that there is inadequate evidence for chronic kidney diseases, bone health and Type 2 diabetes mellitus to formulate recommendations for sodium intake levels to reduce risk of these health outcomes [6]. The increasing body of knowledge on the relationship between sodium intake and a variety of health outcomes will support development of future guidelines on sodium intake.

There were two studies that did not meet our pre-defined criteria for methodological quality but could have potential public health impact. Nasser et al. examined the relationship between BP and estimated sodium intake assessed by 24-h urine and spot urine samples in Bangladesh [42]. Three hundred and thirty-eight participants collected urine for two days, one during the pre-monsoon period and the other over the monsoon season of the same year. They showed a linear association of BP with increased urinary sodium estimated by 24-h urine. In contrast, inverted-V shape plots were observed in both first and second morning spot urine samples. The finding of higher sodium intake being associated with high blood pressure is consistent with previous studies assessing risk of CVD and mortality using 24-h urinary sodium and confirms that spot urine assessments distort the association with health outcomes and are unreliable [15, 84].

Elliot and colleagues investigated the relationship between CVD and mortality and urinary sodium estimated using spot urine samples in the UK Biobank study [25]. The UK Biobank study is a large-scale cohort study aiming to understand the risk of the development of diseases such as cancer, heart disease, stroke, diabetes, and dementia [85]. Participants between 40 and 69 years old provide baseline socio-demographics, lifestyle exposures (including smoking, alcohol, physical activity, and diet); and a range of mental and general health outcomes through questionnaires. They are then followed up through routine health check-ups and the centralised electronic health records of National Health Service. The study included 398 268 participants from the UK Biobank study. Interestingly, the authors reported that they did not find the ‘J’ shape relationship found in other large cohort studies [13, 86] or meta-analyses [87, 88] that used spot urine to measure baseline sodium intake in their analysis. This study found higher risk of stroke and heart failure at extreme intakes of sodium measured using spot urine samples. Given the difficulties (i.e., ethical issues and feasibility to maintain low or high sodium intake) in conducting long-term randomised controlled trials to establish the relationship between sodium intake and health outcomes, the next best evidence is through well-conducted large cohort studies such as the UK Biobank. However, estimating sodium intake using spot urine samples is not a reliable or valid method of assessing sodium exposure as demonstrated by He et al. [15]. Furthermore, spot urine estimates of 24 h urine sodium in the UK Biobank have shown ‘extreme’ intraindividual variability and that disease associations with the estimates of sodium intake are not reproducible [89]. Hence, it is imperative to assess sodium intake via multiple 24-h urine, the most reliable method, as it is not biased by diurnal variation and formulae using sex, age, and body mass index [90]. Albeit with more logistical and participant burden.

Consistent with previous Science of Salt reviews, we found that most RCTs were short-term (≤1month) and assessed intermediate health outcomes (BP) [72–80]. Here, there were no long-term RCTs examining Category I (Mortality) or Category II (Morbidity) outcomes which are considered as being more important to patients. In this review, Category I and II outcomes were studied in cohort studies, where sodium intake was measured with spot urine, despite the fact that an increasing number of studies now confirm that spot urine is an inadequate measure of individual intake [91–93]. Overall few studies (six out of 41 studies) used spot urine as a measurement of sodium intake, while more than half (*n* = 21) of the studies used 24-h urine to determine sodium take. Finally, we found 14 cross-sectional studies examining health outcomes associated with sodium intake. Although these studies are relatively easy to conduct, they are problematic because of the potential for reverse causality and should not be used to establish relationship between sodium intakes and health outcomes [94].

The very high proportion of research identified by the Science of Salt review series that does not meet quality criteria has the potential to undermine integrity of research on dietary sodium. These factors are likely to be contributing to the perceived controversy regarding the health impact of reducing dietary salt and have the potential to undermine public health efforts to reduce dietary salt. Major international and health scientific organisations have provided guidance on minimum criteria for clinical research on dietary sodium sodium which has largely been ignored by funders, researchers, and journals [23, 95]. Journal editors and manuscript reviews need to be cautious in reviewing and accepting manuscript that have methods and designs likely to produce unreliable results.

## Conclusions

This Science of Salt review examined association of sodium intake and health outcomes. Forty-one studies published between September 2019 to December 2020 were identified and two studies met the pre-specified criteria for methodological quality for critical analysis. The two studies examined physical performance and composite renal outcomes. Overall, this review demonstrates further evidence that dietary salt reduction has health benefits and strengthens evidence relating to health outcomes other than BP and CVD. This supports current advice to reduce salt intake which are based on studies examining BP and CVD outcomes but could also have impact on other health outcomes.

## Supplementary information

Supplentary Material

## Data Availability

All data generated or analysed during this study are included in this published article and its supplementary information files.
