# Supplementary material for: The World Hypertension League Science of Salt: a regularly updated systematic review of salt and health outcomes studies (Sept 2019 to Dec 2020)
Source: J Hum Hypertens. 2022 Jun 10;36(12):1048–58. doi: 10.1038/s41371-022-00710-z (PMC9734047; doi:10.1038/s41371-022-00710-z)
Supplement: Supplementary file 1 — Supplentary Material [file 41371_2022_710_MOESM1_ESM.docx]

**Supplementary Table 1**: Hierarchy of outcomes used to select studies for a detailed critical appraisal

| **Type of outcome** | **Examples** |
| --- | --- |
| **Category I**  Mortality reduction | • All-cause mortality  • Disease-specific mortality |
| **Category II**  Morbidity reduction | • Cardiovascular major morbid events  • Stroke  • Other major morbid events (e.g., loss of vision, seizures, fracture, or revascularization)  • Recurrence/relapse/remission of cancer/ disease-free survival  • Renal failure requiring dialysis  • Hospitalizations  • Infections  • Dermatological/rheumatologic disorders |
| **Category III**  Symptoms/ quality of life/functional status | • Quality of life  • Heart failure symptoms  • Symptoms associated with other disorders (e.g. multiple sclerosis)  • Mental illness, depression/anxiety  • Physical function (e.g., 36-Item Short Form Health Survey)  • Headache, migraine |
| **Category IV**  Clinical surrogate outcomes | • Blood pressure  • Diagnosis or prevention of hypertension, osteoporosis, kidney disease not requiring dialysis or other conditions (e.g. diabetes, metabolic syndrome)  • Weight loss or gain considered important to patients  • Renal stones with symptoms  • Memory loss |
| **Category V**  Physiologic surrogate outcomes | • Bone mineral density  • Endothelium-dependent vasodilation  • Markers of the renin-angiotensin-aldosterone  System  • Plasma or urinary norepinephrine  • Novel biomarkers such as marinobufagenin (MBG) excretion  • Heart rate  • Aortic pulse wave velocity  • Carotid artery thickness  • Plasma and urinary nitrate/nitrite  • Uric acid  • Creatinine clearance  • Inflammatory markers  • Blood lipids  • Insulin resistance, blood glucose  • Body composition measures |

**Supplementary Table 2:** Criteria for methodological quality used to select studies for detailed critical appraisal

| **Study design** | **Study quality criteria for inclusion** |
| --- | --- |
| **Systematic reviews and meta-analyses** | - Included observational or experimental studies - Meet criteria outlined for the type of study included |
| **Randomized controlled trials** | - Included at least one group of participants to reduced sodium intake and one group to higher sodium intake (control group) - ≥ 4 weeks duration. - ≥ 40 mmol difference in sodium intake between intervention and control group. - Measured sodium intake using a 24-h urine collection. - The intervention group did not have concomitant interventions (i.e., anti-hypertensive drugs, other dietary interventions) so that the only difference between intervention and control groups must have been sodium intake. |
| **Cohort studies** | - Prospective - Included ≥400 patients (continuous outcomes) or events (dichotomous outcomes) - ≥ 1 year duration - Measured sodium intake for at least 24-h using a 24-h urine collection, food record, or a 24-h food recall |
| **Cross-sectional studies** | - Excluded |

**Supplementary Table 3:** Detailed Risk of Bias assessment of included studies

| **Bias Domain** | **Ratings**  **(High, low, unclear risk)** | **Comments** |
| --- | --- | --- |
| **Study 1: Lana A, Struijk EA, Ortolá R, Rodríguez-Artalejo F, Lopez-Garcia E. Longitudinal Association Between Sodium and Potassium Intake and Physical Performance in Older Adults. The Journals of Gerontology: Series A. 2020;75(12):2379-86.** <https://doi.org/10.1093/gerona/glaa031> | | |
| Sampling | Low | The ENRICA study cohort were selected by stratified cluster sampling. The sample was stratified by province and size of municipality, following which the clusters were randomly selected in two stages (municipalities and census sections). Random telephone dialing was used to select the households in each section. One person from each household was invited to participant. The invited participant was chosen randomly (Rodríguez-Artalejo et al., 2011). |
| Representativeness of sample of underlying population | High | Participants in the ENRICA study were representative of the non-institutionalized population aged 18 years and older in Spain. As noted in the study, “subjects in the households were selected proportionally to the distribution of the population of Spain by sex and age group (18-29, 30-44, 45-64, ≥ 65 years). However, in this current prospective analysis, it was noted that among the 1,130 study participants followed up in 2017, 257 participants were excluded due to lack of dietary data, implausibly high or low energy intakes, and missing anthropometric data. Therefore, it was noted that the participants analyzed in this study “were younger, had higher educational level, better diet quality, less sedentary lifestyle and higher baseline physical performance than the original cohort (2008-2010)”. Therefore, this analysis has limited external validity, as dually noted by the authors: “Seniors-ENRICA cohort was representative of the community dwelling older adult population of Spain, those included in the current longitudinal study sample had better health indicators than the original cohort at baseline. Thereby, the external validity of our findings is not guaranteed and should be addressed by other studies”. |
| Reliability / validity of exposure and outcome measure | Exposure:  High  Outcome:  Low | **Exposure:** Changes in dietary intake of sodium and potassium measured via validated, computer-assisted, face to face diet history. The tool was supplemented with photographs to help participants estimate serving sizes. Standard food composition tables were used to estimate daily sodium and potassium intakes. Among sub-sample of participants diet measurements were evaluated by comparing diet history with 24-h recalls (7) over a 1-years period. Using the residuals method, intakes of minerals were adjusted for total energy intake. Added salt intake was not accounted for, and therefore sodium levels are likely underestimated. Potential for social desirability and memory bias introduced due to the self-reported, retrospective nature of measurement tool.  **Outcome:** Physical performance was measured using the Short Physical Performance Battery, an objective and well-established tool that is valid and reliable to assess physical performance in the elderly. |
| Blinding of outcome assessment | Low | There was no information found regarding blinding of outcome assessment for this study. However, it is unlikely that this would impact study outcomes. |
| Risk of selective outcome reporting | Low | All outcomes were reported in complete form and in usable format. |
| Confounding | Low | Data on potential confounders were collected in the study. Three different models were fitted. Model 1 was age-adjusted. Model 2 was further adjusted for baseline SPPB, sex, educational level, physical activity, and for baseline and changes in BMI, sleep duration, TV-watching, smoker at baseline or during follow-up, alcohol intake, and energy intake. Model 3 adjusted for baseline and incident mortality, including diabetes, hypertension, hypercholesterolemia, cardiovascular (i.e. heart failure, heart attack, and stroke), cancer, musculoskeletal (i.e. arthritis, arthrosis, and hip fracture) diseases. |
| Other comments | - Study was funded by Fondo de Investigaciones Sanitarias grants - Cut offs for recommended intake used in assessment of compliance: Chronic Disease Risk Reduction level for sodium was used and the Adequate Intake level for potassium - Sodium and potassium intake of some participants could have been modified due to health conditions which can impact physical performance, but analyses was adjusted for health outcomes and sensitive analyses were run among people with morbidity. - Potential for reverse causality | |
| **Study 2: Kang M, Kang E, Ryu H, Hong Y, Han SS, Park SK, et al. Measured sodium excretion is associated with CKD progression: results from the KNOW-CKD study. Nephrology Dialysis Transplantation. 2020;36(3):512-9.** <https://doi.org/10.1093/ndt/gfaa107> | | |
| Sampling | High | Non-random sample  From the protocol: “Each participating center will enroll approximately 250 consecutive individuals over a 5-year period from 2011 until 2015…” (Oh et al 2014) |
| Representativeness of sample of underlying population | Unclear | Not enough information provided to assess representativeness |
| Reliability / validity of exposure and outcome measure | Exposure:  Low  Outcome:  Low | Measured 24-hour urinary sodium to assess dietary salt intake; excluded if 24-hour urine samples were <500mL/day  Composite renal outcome, defined as eGFR halving or developing end-stage renal disease. The former was calculated using an equation developed by the CKD Epidemiology Collaboration. The latter was defined as initiation of dialysis or kidney transplantation. |
| Blinding of outcome assessment | Low | Blinding of outcome assessment was not mentioned in the paper, but unlikely to affect outcomes. |
| Risk of selective outcome reporting | Low | Outcome measures reported in a complete and usable format. Reasons for excluding participants from the complete-case analysis were described. |
| Confounding | Low | Cox proportional hazard models were fitted, adjusted for: age, sex, BMI, baseline urinary creatinine (Model 1); urinary potassium excretion, baseline eGFR (Model 2); CKD etiology, presence of diabetes mellitus, use of diuretics, use of RAS blockers, urinary protein excretion and SBP (Model 3) |
| Other comments | - Study funded by the Korea Centers for Disease Control and Prevention (KCDC) - Large prospective cohort study including CKD patients with long follow-up - Subgroup analyses comparing Q4 versus Q2 of sodium excretion were conducted by: age, sex, baseline eGFR, presence of uncontrolled hypertension, diabetes, obesity, use of RAS blockers, use of diuretics, 24-hour urinary potassium, and presence of proteinuria - Sensitivity analysis was conducted using spot urine (Tanaka equation)   Sodium excretion was also analysed as a continuous variable (per 100mEq/day) – results reported in the supplementary file | |

**References Supplementary Table 3:**

Oh K-H, Park SK, Park HC, Chin HJ, Chae DW, Choi KH, et al. KNOW-CKD (KoreaN cohort study for Outcome in patients With Chronic Kidney Disease): design and methods. BMC Nephrol. 2014;15:80.

Rodríguez-Artalejo, F., Graciani, A., Guallar-Castillón, P., León-Muñoz, L. M., Zuluaga, M. C., López-García, E., Gutiérrez-Fisac, J. L., Taboada, J. M., Aguilera, M. T., Regidor, E., Villar-Álvarez, F., & Banegas, J. R. (2011). Justificación y métodos del estudio sobre nutrición y riesgo cardiovascular en España (ENRICA) [Rationale and methods of the study on nutrition and cardiovascular risk in Spain (ENRICA)]. *Revista espanola de cardiologia*, *64*(10), 876–882. <https://doi.org/10.1016/j.recesp.2011.05.019>
